# Associations of Salivary Microbiota with Diet Quality, Body Mass Index, and Oral Health Status in Turkish Adolescents

**DOI:** 10.3390/nu17213434

**Published:** 2025-10-31

**Authors:** Büşra Aslan Gönül, Ebru Delikan, Betül Çiçek, Meral Yılmaz Cankılıç

**Affiliations:** 1 Department of Nutrition and Dietetics, Institute of Health Sciences, Erciyes University, Kayseri 38280, Türkiye; busra.aslan@erciyes.edu.tr; 2 Department of Pediatric Dentistry, Faculty of Dentistry, Nuh Naci Yazgan University, Kayseri 38170, Türkiye; e.delikan@gmail.com; 3 Department of Biology, Faculty of Science, Eskişehir Technical University, Eskişehir 26555, Türkiye; meralyilmaz@eskisehir.edu.tr

**Keywords:** oral microbiota, 16S rRNA sequencing, nutrition, oral health, dental caries, periodontal status

## Abstract

**Background:** The oral microbiota is the largest and most diverse microbial community in the human body, shaped by numerous factors such as body composition, dietary habits, and oral health status. However, relationships between these parameters and the salivary microbiota in adolescents are not yet well understood. **Objectives:** This study aimed to characterize the salivary microbiota of healthy Turkish adolescents and to examine its associations with body mass index (BMI), diet quality, decayed-missing filled teeth (DMFT) index, and community periodontal index of treatment needs (CPITN). **Methods:** A descriptive, cross-sectional study was conducted among 40 adolescents aged 14–18 years, classified into four BMI z-score categories (underweight, normal weight, overweight, and obese). Anthropometric measurements, nutritional information, and oral health parameters (DMFT, CPITN) were assessed. Unstimulated saliva samples were collected, and the V3–V4 region of the 16S rRNA gene was sequenced using Illumina MiSeq. Alpha and beta diversity, differential abundance (LEfSe), and correlations with HEI components were analyzed. **Results:** Underweight adolescents exhibited significantly higher alpha diversity than obese participants (*p* = 0.024), while beta diversity did not differ across BMI, HEI, DMFT, or CPITN categories. LEfSe analysis revealed BMI-specific taxa: *Leptotrichia* sp., *Haemophilus* sp., and *Treponema socranskii* were enriched in the underweight group; *Prevotella denticola* in the obese group; and *Selenomonas sputigena* in the normal-weight group. HEI components, including whole fruits, whole grains, and plant-based proteins, showed positive correlations with *Desulfobacterota* and *Proteobacteria*. Poor oral hygiene was associated with higher species richness but not with large shifts in community structure. **Conclusions:** Salivary microbiota diversity and composition in adolescents vary with BMI, diet quality, and oral hygiene. These patterns are consistent with the idea that targeted nutritional and oral health interventions could influence the salivary microbiome during adolescence.

## 1. Introduction

The oral microbiome is the second largest and most diverse microbial community in the human body after the gut [1]. The acquisition and establishment of the oral microbiota is a dynamic process that begins prenatally, continues during birth, and develops postnatally through interactions with caregivers, diet, and the environment. These stages shape the oral microbial ecosystem, which further evolves during the transition from deciduous to permanent dentition and throughout adolescence [2]. It is present in both saliva and the surfaces within the oral cavity. The salivary bacteriome is predominantly composed of the genera *Streptococcus*, *Prevotella*, and *Veillonella*, which together account for approximately 70% of the salivary microbiota [3]. In a healthy state, diverse bacterial species coexist in balance. Disruption of this balance facilitates the proliferation of pathogenic taxa such as *Porphyromonas gingivalis*, *Tannerella forsythia*, and *Treponema denticola*, contributing to periodontal disease and potential tooth loss [4].

Diet substantially influences oral microbial composition. Fermentable carbohydrates, particularly sucrose, promote acid production by species such as *Streptococcus sanguis* and *Streptococcus mutans*, altering community structure and enhancing plaque formation [5]. Although dietary fats are not digested orally, saliva contains various lipids, including fatty acids, cholesterol, and triglycerides. Dietary fats can also influence food texture, potentially reducing oral retention time and tooth adhesion. Additionally, micronutrient intake has been associated with variations in the oral microbiota [1]. While associations between diet quality and oral health are well established [6,7], evidence linking diet quality to the oral microbiota remains limited.

The oral microbiome contributes to protein digestion, carbohydrate deglycosylation, and sulfate reduction, and its anaerobic components may play a role in the prevention and management of metabolic disorders [8]. Poor oral health has been associated with an increased risk of obesity [9]. Although the potential relationship between oral microbiota and obesity has attracted research interest in children, adolescents, and adults [10,11,12], it is not yet fully understood.

Geographic factors may also influence the composition of the oral microbiota, highlighting the need for research in diverse populations to better understand regional variations [13]. To date, no studies have investigated the oral microbiota of healthy adolescents in Türkiye.

Therefore, the present study aimed to characterize the salivary microbiota of healthy Turkish adolescents and examine its associations with body mass index (BMI), diet quality, decayed-missing-filled teeth (DMFT) index, and community periodontal index of treatment needs (CPITN). The study was designed to test the following hypotheses:

- Salivary microbiota diversity and composition differ across BMI z-score categories (underweight, normal weight, overweight, and obese adolescents).
- Diet quality, assessed by the Healthy Eating Index (HEI), is associated with salivary microbial diversity and specific bacterial taxa.
- Oral health status (DMFT, CPITN) is associated with differences in salivary microbiota diversity and community structure.

## 2. Materials and Methods

This descriptive and cross-sectional study was approved by the Erciyes University Non-Interventional Clinical Research Ethics Committee (Approval No: 2023/839 Date: 20 December 2023). All procedures complied with the Declaration of Helsinki, and written informed consent was obtained from the parents or guardians of all participants prior to the study.

### 2.1. Participants

The World Health Organization (WHO) classifies individuals aged 12–18 years as adolescents. However, in the present study, individuals aged 12–13 years were excluded due to the ongoing eruption of permanent canines [14]. The study specifically focused on adolescents aged 14–18 years to investigate the oral microbiota during the period of established permanent dentition [15,16]. A total of 40 participants (10 underweight, 10 normal weight, 10 overweight, and 10 obese) were enrolled from a family health center in Kayseri, Türkiye. Inclusion criteria encompassed the absence of special medical nutritional therapy, chronic systemic/nutritional/endocrine disorders, and congenital developmental anomalies. Exclusion criteria included the presence of severe oral disease, a history of periodontal treatment (e.g., supragingival curettage or root planing), use of antibiotics, anti-inflammatory agents, or sedatives within the past three months, and tobacco use.

### 2.2. Study Design

**Figure 1:**
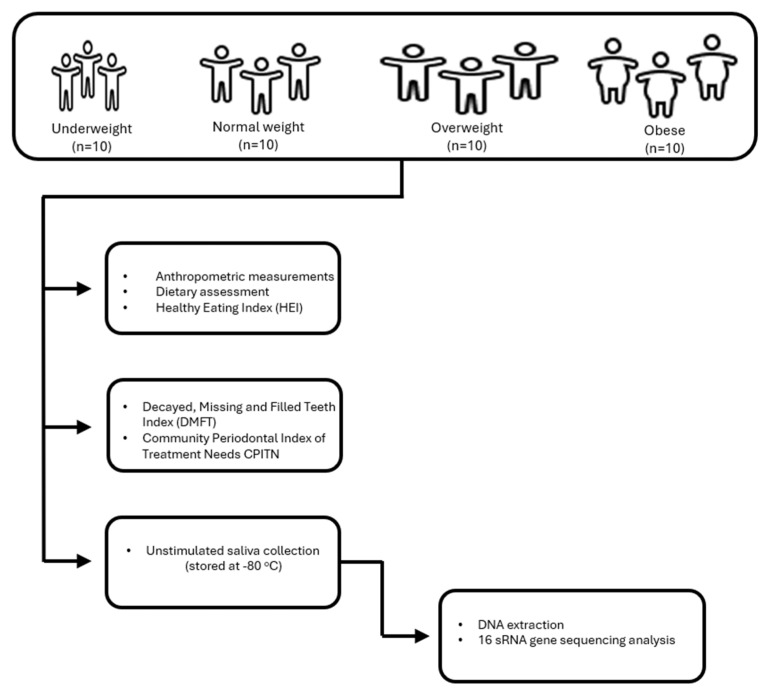
Flowchart for the inclusion of participants in the study.

Participants’ anthropometric measurements, dietary assessments, and Healthy Eating Index (HEI) data were collected through structured questionnaires and face-to-face interviews conducted by a trained researcher (B.A.). All intraoral examinations were performed by a single pediatric dentist (E.D.) using a dental mirror and probe under natural daylight near a window, supplemented by headlamp illumination. For oral microbiota analyses, saliva samples were obtained from children who had not brushed their teeth or consumed any food since the morning. Samples were immediately stored at −80 °C until analysis. They were transferred to the analysis center under cold chain conditions, and DNA isolation and sequence analysis were performed together with molecular biologists. The flowchart for the inclusion of participants in the study is given in Figure 1.

### 2.3. Anthropometric Measurements

A trained researcher conducted all anthropometric assessments using standard protocols. Body weight and fat percentage were measured using a bioelectrical impedance analyzer (TANITA BC 532, Tanita Corp., Tokyo, Japan) while participants were wearing light clothing and no shoes [17]. Height was measured with a stadiometer in the Frankfurt plane with the feet side-by-side, head, hips, and heels touching the wall. Waist circumference was measured with a non-stretchable tape measure passing through the midpoint between the lowest rib and the crista-iliac region, with the arms at the sides and feet together. BMI was calculated as weight (kg) divided by height squared (m^2^) and evaluated according to the WHO 5–19 age BMI z-score classification. The WHO AnthroPlus v1.0.4 software was used to calculate the z-score values of the children [18]. According to the BMI z score, participants were classified as underweight (UW, ≤−1 SD), normal (NW, −1 to +1 SD), overweight (OW, +1 to +2 SD), or obese (OB, ≥+2 SD) [19].

### 2.4. Dietary Assessment

Dietary intake of adolescents was evaluated using a three-day food record. The first day of the food consumption record was recorded by the researcher in a 24-h dietary recall. Data for subsequent days were collected through phone interviews. The three-day food consumption averages of the participants were entered into the BeBiS Nutrition Information System (version 8.2) software, and three-day energy (kcal) and macro- and micronutrient intakes were calculated.

### 2.5. Healthy Eating Index

The Healthy Eating Index (HEI) was calculated using the three-day dietary data. The HEI comprises 10 components (total fat, saturated fat, cholesterol, fruit, vegetables, grains, milk, meat, sodium, and food variety), each scored from 0 to 10. The index score rises with increased consumption of dietary components that should be consumed adequately but falls as consumption of components that require limitation grows. The maximum score is 100, with a score of ≤50 deemed “poor,” 51–80 indicating “needs improvement,” and ≥80 classified as “good” [20].

### 2.6. Oral Health Assessment

#### 2.6.1. The Decayed, Missing, and Filled Teeth Index (DMFT)

During the clinical assessment, a comprehensive evaluation of the oral cavity’s soft and hard tissues, the temporomandibular joint, and occlusal relationships was conducted. Dental caries experience was assessed using the DMFT index, a widely accepted measure of caries prevalence and severity developed by Klein, Palmer, and Knutson in 1938 [21]. DMFT scores were categorized into five levels according to WHO guidelines: very low (0.0–1.1), low (1.2–2.6), moderate (2.7–4.4), high (4.5–6.5), and very high (>6.6) [22]. In this study, DMFT scores were used as low (≤2.6), moderate (2.7–4.4), and high (≥4.5).

#### 2.6.2. Community Periodontal Index of Treatment Needs (CPITN)

Periodontal status was evaluated using CPITN, with a WHO probe applying 20–25 g force [23]. Ten index teeth (1.6/1.7/1.1, 2.6/2.7, 3.6/3.7/3.1, and 4.6/4.7) were examined; if missing, all teeth in the sextant were assessed. Periodontal status was recorded based on the most severe condition observed in each sextant, using the following coding system: Code 0 indicated healthy periodontal tissue; Code 1 represented bleeding on probing; Code 2 denoted the presence of supra- or subgingival calculus and plaque; Code 3 corresponded to a pocket depth of 4–5 mm; Code 4 indicated a pocket depth of 6 mm or more; and X was used when no teeth were present or the sextant could not be assessed.

According to these codes, the periodontal status was scored, and the need for treatment was determined. Treatment need scores (TNs) ranged from 0 to 4 and were based on the most severe periodontal status of all teeth. In this way, TN0 indicates that periodontal treatment is not required when the gums are healthy (code 0), TN1 indicates that oral hygiene (OH) should be improved (code 1); TN2 indicates that tooth surface cleaning, removal of overflow fillings and improvement of OH (codes 2+3) are needed; and TN3 indicates that further treatment is required (code 4). In the current study, TN0 was evaluated as good OH, and TN1, TN2, TN3, and TN4 were evaluated as poor OH.

No radiographic imaging or invasive procedures were performed. Participants were informed about their oral health status and referred to a dentist if necessary.

### 2.7. Saliva Sampling, DNA Extraction, Sequencing, and Bioinformatics

To minimize the effect of circadian rhythm, saliva samples were collected between 8–11 am from children who had not brushed their teeth and had not consumed any food since the morning hours. Unstimulated saliva samples (5 mL) were collected directly into sterile tubes (Isolab, İstanbul, Türkiye) and stored at −80 °C. All samples were shipped overnight on dry ice packs to Diagen Biotechnology Systems Corp. (Ankara, Türkiye) for DNA extraction. After thawing, 9 mL Phosphate-Buffered Saline (PBS) was added, centrifuged at 400× *g* for 10 min, and the pellet processed for DNA extraction using the QuickGene DNA Tissue kit S (Kurabo, Industries Ltd., Osaka, Japan) according to the manufacturer’s instructions. Protocol steps included lysis with Proteinase K, ethanol precipitation, washing, and elution, yielding ~50–60 ng genomic DNA.

The V3–V4 hypervariable region of the 16S rRNA gene was amplified through polymerase chain reaction (PCR). The PCR protocol consisted of an initial denaturation at 95 °C for 3 min, followed by 25 cycles of denaturation at 55 °C for 30 s, annealing at 52 °C for 30 s, and elongation at 72 °C for 30 s, with a final extension at 72 °C for 5 min. The resulting PCR amplicons were separated via 2% agarose gel electrophoresis and purified using the Agencourt AMPure XP system (Beckman Coulter Genomics, Brea, CA, USA). Library preparation was performed, and sequenced on a MiSeq system (Illumina Inc., San Diego, CA, USA).

Paired-end Illumina reads (2 × 250 bp) were imported into the QIIME2 environment (version 2023.7) [24]. All samples achieved a sequence depth exceeding 100×, and no samples were excluded. Quality filtering, chimera detection, and read trimming were performed using the QIIME2 DADA2 pipeline (q2-dada2), with bases below a Phred quality score of Q30 removed [25]. Amplicon Sequence Variants (ASVs) generated by DADA2 were taxonomically classified against the SILVA 138 (https://www.arb-silva.de/documentation/release-138/ accessed on 1 March 2025) database [26,27]. A Phyloseq object was constructed from QIIME2 artifacts in R (version 4.1) for downstream analysis [28,29]. Alpha diversity was assessed using Chao1, Shannon, and Simpson indices to evaluate within-sample taxonomic diversity [30]. Beta diversity, reflecting taxonomic differences between samples, was calculated using Jaccard, Bray–Curtis, weighted UniFrac, and unweighted UniFrac distances. Differential abundance analysis was performed using the DESeq2 R package v1.40.2 to identify significant taxonomic differences between groups [31]. Linear discriminant analysis Effect Size (LEfSe) analysis was conducted between groups to display statistically significant taxonomies [32].

### 2.8. Statistical Analysis

Statistical analyses were performed using the IBM Statistical Package for Social Sciences (SPSS), version 27.0. Categorical variables were expressed as frequencies and percentages, and group comparisons were conducted using Chi-square tests. The normality of continuous variables was assessed by the Shapiro–Wilk test, and visual inspection was supported with descriptive plots (e.g., histograms). For normally distributed variables, means and standard deviations were calculated; differences between groups were analyzed using independent samples t-tests or one-way analysis of variance (ANOVA). For microbiota data, non-parametric Kruskal–Wallis tests were applied. A *p*-value < 0.05 was considered statistically significant.

## 3. Results

### 3.1. Characteristics of the Participants

Table 1 presents the demographic and socioeconomic characteristics of all participants and subgroups categorized by BMI z-score classification.

The study included 40 adolescents with a mean age of 15.0 ± 0.7 years. The mean waist circumference was 74.5 ± 13.2 cm, and the mean body fat percentage was 24.4 ± 10.6%. These parameters were significantly higher in obese participants compared to other groups (*p* < 0.001). More than half of the participants were female (57.5%). The majority of their mothers and fathers had completed at least a high school education (77.5% and 62.5%, respectively). There were no significant differences among BMI groups in terms of age (*p* = 0.837), gender distribution (*p* = 0.771), or parental educational status (maternal: *p* = 0.104; paternal: *p* = 0.759). However, waist circumference and percentage of body fat differed significantly across the BMI categories (*p* < 0.001 for both). Post hoc comparisons indicated that mean waist circumference and body fat percentage increased consistently from the underweight to the obese groups. Specifically, obese adolescents had the highest waist circumference (90.1 ± 8.4 cm) and body fat percentage (36.5 ± 5.8%), while underweight adolescents had the lowest values for both variables (60.9 ± 4.9 cm and 12.3 ± 5.1%, respectively).

### 3.2. Oral Health

Table 2 summarizes oral health, OH, and oral health behaviors among participants and BMI-based subgroups. Tooth brushing frequency, brushing duration, frequency of dental visits, DMFT, CPITN, and deviation did not differ across BMI categories (*p* > 0.05). Overall, 32.5% of the adolescents reported brushing their teeth either sometimes or once per day, while only 7.5% reported brushing three times a day.

The majority of participants reported brushing for 2–3 min (62.5%). Additionally, 82.5% visited the dentist only in the presence of dental problems, 60.0% had a low DMFT index, 65.0% demonstrated poor OH, and 72.5% exhibited Class I occlusion. Among adolescents with normal body weight, more than half (60.0%) had Class II occlusion, whereas Class I occlusion was more prevalent in the other BMI groups (*p* = 0.035).

### 3.3. Amplicon Sequencing Data Analyses

Following quality filtering and preprocessing steps, a total of 5,187,474 high-quality reads were obtained, with a mean of 129,687 reads per sample. A total of 2316 operational taxonomic units (OTUs) were identified at a 97% sequence similarity threshold. OTU counts by group were as follows: obese (626), overweight (551), underweight (588), and normal weight (551). These OTUs were taxonomically assigned to 16 phyla, 27 classes, 64 orders, 89 families, and 157 genera.

**Figure 2:**
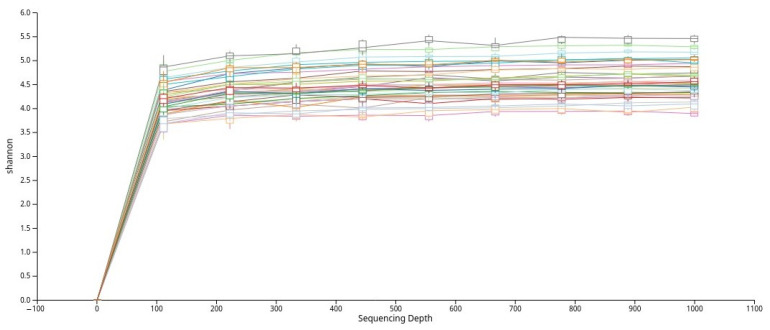
Rarefaction curves based on the Shannon diversity index that show adequate sequencing depth across all samples.

To evaluate alpha diversity, the dataset was rarefied, and rarefaction curves based on the Shannon index were generated (Figure 2). The curves plateaued across all samples, indicating that the sequencing depth was sufficient to capture the majority of microbial diversity present in the saliva samples.

### 3.4. Alpha Diversity Analyses

**Figure 3:**
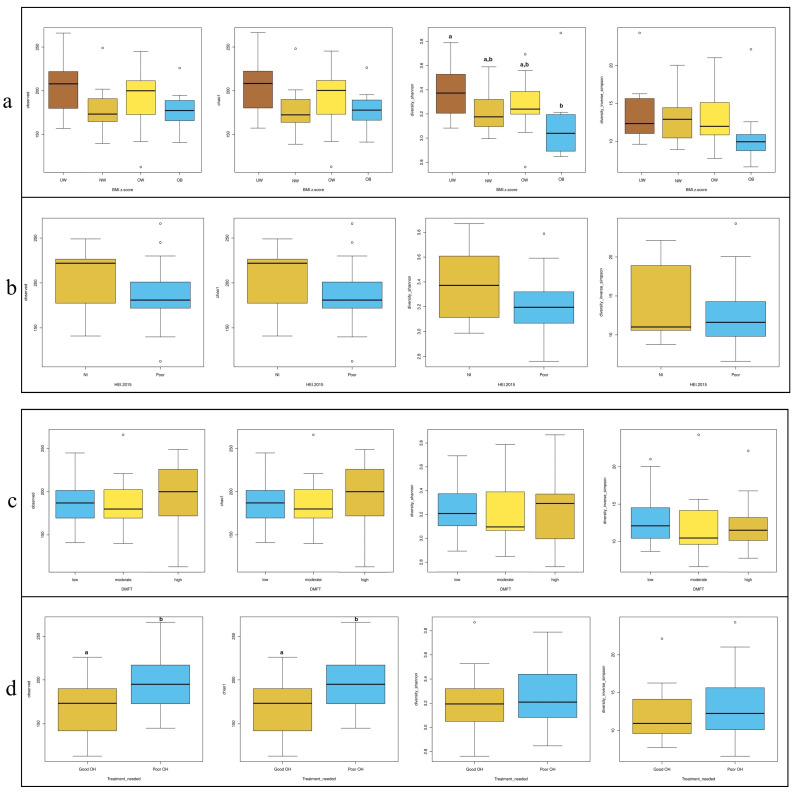
Distribution of alpha diversity metrics (Observed OTUs, Chao1, Shannon, and In-verse-Simpson) across groups stratified by (**a**) BMI z-score, (**b**) HEI, (**c**) DMFT, and (**d**) treatment needed. Different letters (a, b) denote significant differences (*p* < 0.05).

In Figure 3, four commonly used alpha diversity indices—Observed OTUs, Chao1, Shannon, and Inverse Simpson—were calculated to assess bacterial diversity and richness across subgroups based on BMI z-score, HEI, DMFT score, and periodontal treatment need. According to BMI z-score classification (Figure 3a), the underweight (UW) group exhibited significantly higher species diversity compared to the obese group (*p* = 0.024). Regarding treatment need (Figure 3d), individuals with poor OH had significantly higher diversity scores than those with good OH based on the Observed OTUs and Chao1 indices (*p* = 0.025 for both). No significant differences in alpha diversity were observed across HEI (Figure 3b) or DMFT (Figure 3c) groups.

### 3.5. Beta Diversity Analyses

**Figure 4:**
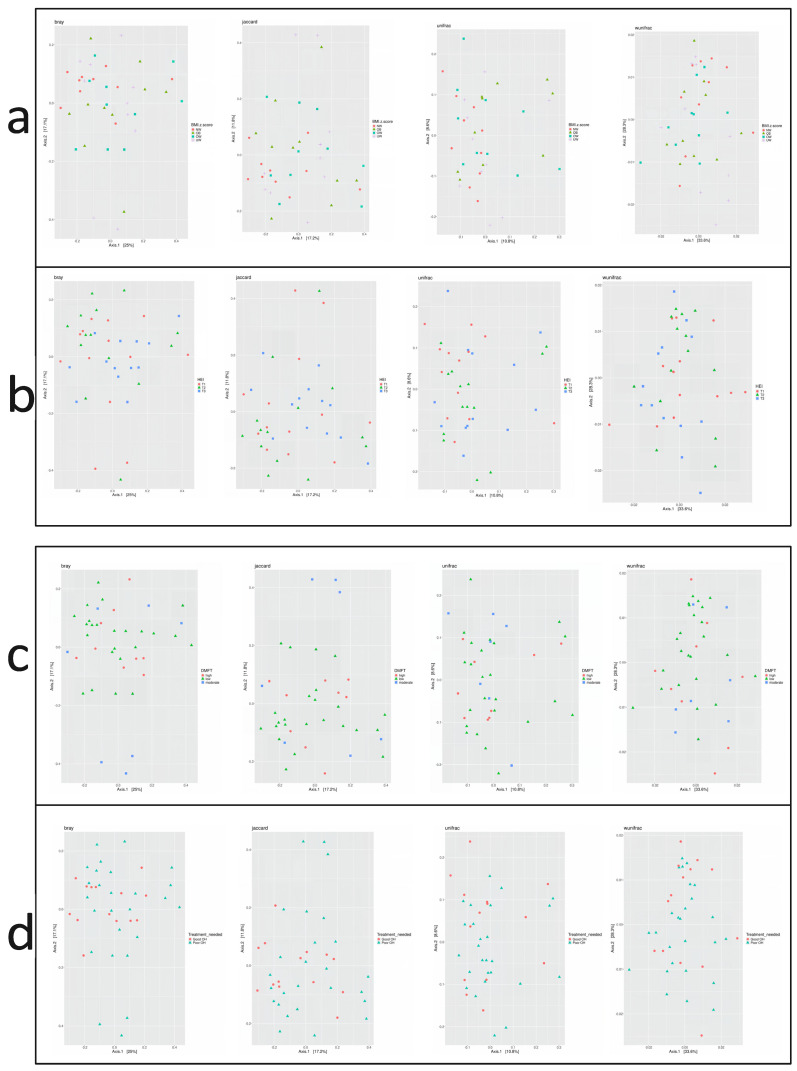
Principal Coordinate Analysis (PCoA) plots based on beta diversity metrics: Bray–Curtis, Jaccard, Unweighted UniFrac, and Weighted UniFrac. Beta diversity is visualized across groups stratified by (**a**) BMI z-score, (**b**) Healthy Eating Index (HEI), (**c**) DMFT score, and (**d**) treatment need.

Beta diversity describes the variation in microbial composition between samples or groups and reflects similarities or differences in species presence and abundance. In the current study, four beta diversity metrics were applied: Bray–Curtis, Jaccard, Unweighted UniFrac, and Weighted UniFrac. As shown in Figure 4, no significant differences in beta diversity were observed across groups defined by BMI z-score, HEI score, DMFT score, or periodontal treatment need (*p* > 0.05 for all metrics). Microbial community composition did not vary substantially between these subgroups.

### 3.6. LEfSe Analysis

**Figure 5:**
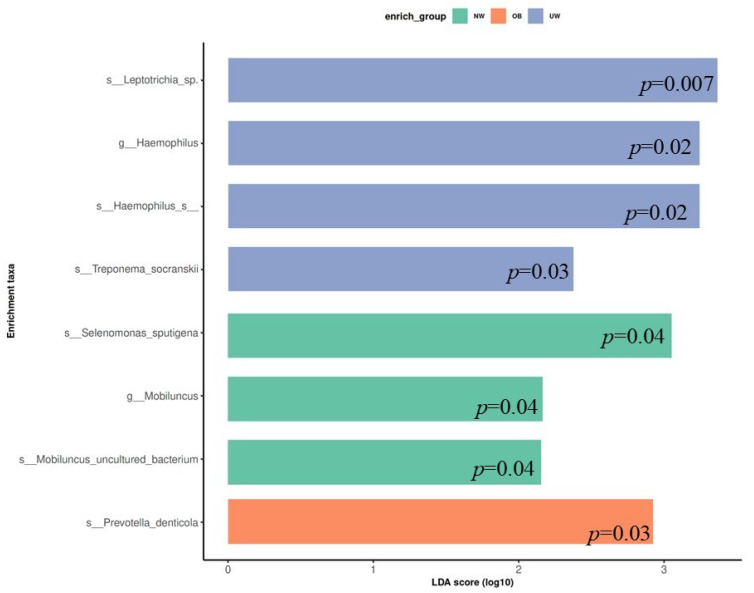
Linear Discriminant Analysis Effect Size (LEfSe) plot showing differentially abundant bacterial taxa across BMI z-score groups. Only taxa with LDA scores > 2.0 are shown.

To identify bacterial taxa that were differentially abundant among BMI z-score groups, LEfSe (Linear Discriminant Analysis Effect Size) was performed. The analysis revealed that specific microbial taxa were significantly enriched in each BMI category (LDA score > 2.0, *p* < 0.05). In the UW group, higher relative abundances were observed for *Leptotrichia* sp., *Haemophilus* sp., *Treponema socranskii*, and *Haemophilus* sp. (*p* = 0.007, 0.02, 0.03, and 0.02 respectively). The normal weight (NW) group exhibited significant enrichment of *Selenomonas sputigena*, *Mobiluncus* uncultured bacterium, and the genus *Mobiluncus* (*p* = 0.04, 0.04, and 0.04 respectively). In contrast, *Prevotella denticola* was significantly more abundant in the obese (OB) group (*p* = 0.03) (Figure 5).

### 3.7. Taxonomic Composition

**Figure 6:**
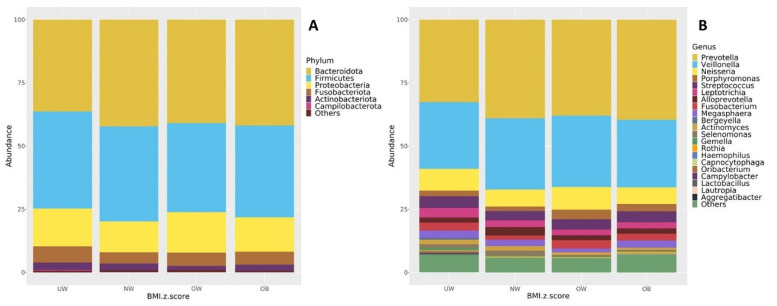
Taxonomic composition of the oral microbiota (≥1% relative abundance) shown at the (**A**) phylum level and (**B**) genus level across BMI z-score groups.

The core microbiome composition of each BMI group was examined at both the phylum and genus levels. Figure 6 presents taxa bar plots illustrating the relative abundance of dominant microbial groups (≥1%) across BMI z-score categories. At the phylum level (Figure 6A), the most abundant phyla across the UW, NW, OW, and OB groups were *Bacteroidota* (37.1%, 42.2%, 40.1%, 41.1%), *Firmicutes* (38.8%, 36.6%, 33.9%, 37.2%), and *Proteobacteria* (15.0%, 13.0%, 17.8%, 13.0%), respectively. Other detected phyla included *Fusobacteria, Actinobacteriota*, and *Campylobacterota*, each accounting for smaller proportions. Bacteroidota were the main abundant phylum in the NW, OB, and OW groups. *Bacteroidota* and *Firmicutes* were both abundant in the UW group.

At the genus level (Figure 6B), all BMI groups were predominantly composed of *Prevotella* (33.2%, 38.8%, 36.9%, 39.0%), *Veillonella* (26.6%, 27.4%, 27.6%, 27.4%), and *Neisseria* (8.8%, 7.2%, 10.2%, 6.2%), respectively. The fourth most abundant genus was *Streptococcus* in the UW, NW, and OB groups (4.9%, 3.6%, and 4.5%, respectively), while *Porphyromonas* ranked fourth in the OW group (4.2%).

### 3.8. Heatmap Analysis of Salivary Microbiota

**Figure 7:**
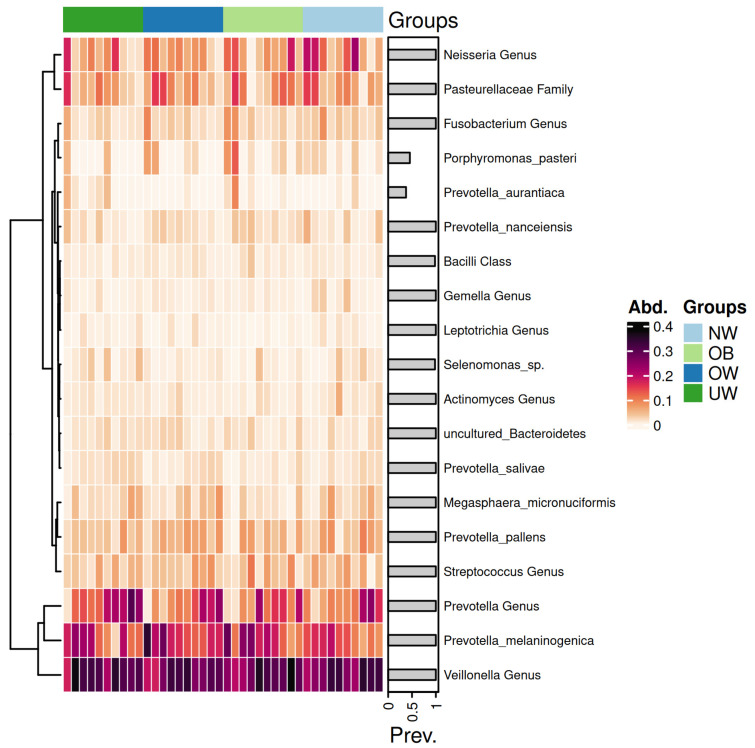
Heatmap of saliva microbiota abundance across BMI z-score groups. Each row represents a taxonomic unit, and each column corresponds to an individual sample. Color intensity reflects the relative abundance of each taxon, with darker shades indicating higher levels. The groups are ordered from left to right as underweight (UW), overweight (OW), obese (OB), and normal weight (NW).

Figure 7 presents a heatmap displaying the relative abundance of dominant bacterial genera and species across BMI z-score groups.

The genera *Prevotella* and *Veillonella* exhibited consistently high abundance across all BMI categories, forming the core of the salivary microbiome. Notably, *Prevotella melaninogenica* was particularly abundant in the OB and OW groups. In contrast, the UW and NW groups showed a higher relative abundance of *Neisseria* species. Notably, the *Prevotella* genus exhibited a higher abundance in the UW group compared to the other groups. Similarly, the *Streptococcus* genus showed elevated levels in the OB group, while its abundance decreased progressively in the OW, NW, and UW groups. In contrast, genera such as *Leptotrichia* demonstrated consistently low abundance across all groups, with minimal variation. The *Neisseria* genus appeared more prevalent in the NW group, suggesting a potential association with a balanced BMI.

### 3.9. Correlation Between Microbiota and HEI

**Figure 8:**
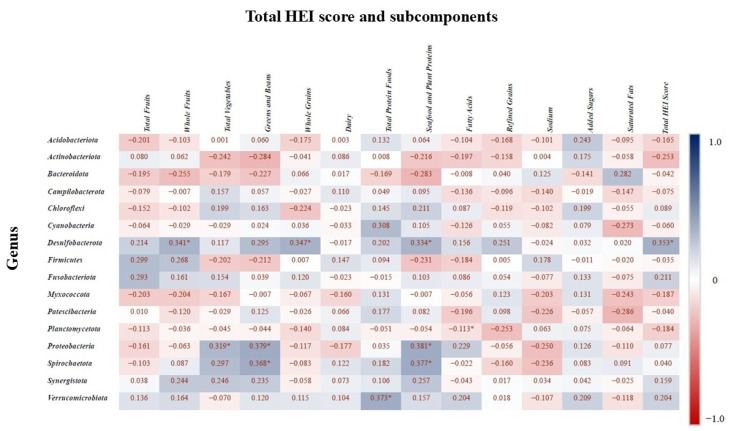
Spearman correlation values indicate the relationship between bacterial genera and the HEI components. Blue indicates the strongest positive correlations, and red indicates the strongest negative correlations. “*” indicates statistically significant correlation (*p* < 0.05).

Spearman correlation analysis was conducted to assess the relationship between the relative abundances of bacterial phyla and components of the HEI subcomponents (*p* < 0.05 with asterisks). Figure 8 presents the correlation matrix, where positive correlations indicate higher total HEI scores and subcomponents are associated with increased relative abundance of certain taxa, while negative correlations indicate a decrease in abundance. The phylum *Desulfobacterota* showed a positive correlation with whole fruit intake, whole grain intake, seafood and plant protein intake, and the total HEI score. *Planctomycetota* was positively correlated with fatty acid intake, while *Proteobacteria* was positively associated with total vegetable intake, greens and beans, and seafood and plant proteins. Additionally, *Verrucomicrobiota* abundance was positively correlated with total protein food intake, and *Spirochaetota* was associated with consumption of greens and beans.

## 4. Discussion

This study investigated the relationship between salivary microbiota composition and multiple health–related parameters—namely BMI, dietary quality, and oral health status—in adolescents aged 14-to-18 years. The findings indicated that microbial diversity and taxonomic profiles varied across BMI groups. Specifically, UW adolescents exhibited significantly higher alpha diversity compared to their OB counterparts. Differential abundance analysis identified distinct microbial taxa enriched in each BMI category. Diet quality, as assessed by the HEI, showed positive associations with several bacterial phyla, suggesting that nutritional intake may be linked to oral microbial ecology. Moreover, adolescents with poor OH presented greater species richness than those with good hygiene, although overall microbial community structure remained stable. Collectively, the findings suggest that BMI, dietary patterns, and oral hygiene are each associated with specific shifts in salivary microbiota. Therefore, the study’s hypotheses regarding these associations were supported.

Interest in exploring the salivary microbiota is increasing due to its potential role in human health and disease [33]. The oral cavity is home to more than 700 bacterial species. A pH of 6.5–7.5 and a temperature of 37 °C provide conditions that allow pathogenic and mutualist bacteria to coexist [34]. The dominant bacterial phyla observed in the salivary microbiota—*Bacteroidota*, *Firmicutes*, and *Proteobacteria*—are consistent with previous studies in adolescents [35,36]. Our study detected significantly higher alpha diversity in the underweight (UW) group than in the obese (OB) group. This observation is consistent with prior research indicating that obesity may be associated with decreased oral microbial diversity due to altered metabolic and immune responses [10,12].

An additional observation was the distribution of malocclusions, whereby normal-weight adolescents exhibited a relatively higher prevalence of Class II malocclusion compared with the other BMI groups. The mechanisms underlying this pattern remain uncertain; however, evidence from the orthodontic literature indicates that occlusal conditions and orthodontic treatment have been associated with oral mucosal alterations, which may, in turn, influence microbial balance. Manuelli et al. [37] reported that orthodontic treatment could be linked to oral mucosal complications, that potentially affect oral health status. Although present study did not directly investigate orthodontic factors, acknowledging their potential influence provides an additional clinical perspective when interpreting salivary microbiota findings in adolescents [37].

LEfSe analysis identified distinct taxa characterizing each BMI category. *Leptotrichia* sp., *Haemophilus* sp., and *Treponema socranskii* were more abundant in the UW group, while *Prevotella denticola* was relatively enriched in the OB group. Elevated levels of *Prevotella* species, often associated with inflammation and carbohydrate-rich diets, have previously been documented in both oral and gut microbiota of individuals with obesity [11,38]. In contrast, *Haemophilus* species—frequently linked to oral health and immune modulation—were more abundant in underweight participants, which may reflect less inflamed microbial environment. It should be noted, however, that these differences may also reflect oral hygiene practices or other environmental factors rather than BMI per se, and therefore the associations should be interpreted with caution. Further studies are needed to clarify the specific role of these taxa in relation to body weight and metabolic status.

Beta diversity metrics did not differ significantly between BMI groups, suggesting that BMI influences species richness rather than the overall structure of the microbiota. This observation is in line with reports by Meslier et al. [39] in gut microbiota studies, where differences were more evident at the level of specific taxa than in broader community composition.

Unlike most prior studies that focused on specific nutrient components [1,40] or diet quality [41,42], our study examined association between HEI scores and microbial taxa. To the best of our knowledge, this is the first study to investigate the salivary microbiota in relation to HEI and its subcomponents in adolescents aged 14–18 years. Positive associations were observed between the abundance of *Desulfobacterota* and higher intake of whole fruits, whole grains, and seafood and plant proteins. Although *Desulfobacterota* have not been previously studied in the oral cavity, their presence in the gut has been linked to low-carbohydrate diets [43,44]. Similarly, *Proteobacteria* abundance showed positive associations with total vegetable intake, greens, beans, seafood, and plant-based proteins. *Proteobacteria*, including the genera *Neisseria* and *Haemophilus*, common members of the oral microbiota of healthy individuals [45]. Existing evidence suggests their abundance may increase with plant-based diets and decrease with Western-style, low-fiber diets [46]. In line with this, short-term application of plant-based diets has been associated with an increase in the relative abundance of *Proteobacteria* in the oral microbiome [47]. An increase *Proteobacteria* has also been observed in the gut microbiome of mice fed plant protein sources [48]. However, an other study reports inconsistent results—for example, a 16-week vegan diet was associated with a decrease in *Proteobacteria* abundance [49]. Thus, although *Proteobacteria* are generally dominant in the oral microbiome of healthy individuals, findings regarding their dietary associations remain limited and sometimes contradictory [46]. Our findings also pointed to a positive association between total protein food intake and *Verrucomicrobiota* abundance. This phylum includes *Akkermansia muciniphila*, a species known for maintaining epithelial barrier function and commonly found in the gut [46]. While no previous studies have directly examined *Verrucomicrobiota* in relation to diet quality in the salivary microbiota, our findings are consistent with evidence showing that omnivores tend to exhibit a higher relative abundance of *Verrucomicrobiota* in the gut microbiota compared to vegans [44].

Alpha diversity indices (Observed OTUs and Chao1) indicated that adolescents with poor OH had significantly higher species richness compared to those with good hygiene. This pattern may be related to increased microbial colonization associated with insufficient oral care, encompassing both commensal and pathogenic species. These observations are consistent with previous studies by Hall et al. [50] and Bertelsen et al. [51], which reported that poor OH was associated with elevated microbial load and diversity, contributing to gingival inflammation. Hall et al. [50] systematically examined bacterial shifts during the development and resolution of gingival inflammation across four oral sites, noting a decline in *Streptococcus*, *Neisseria*, and *Actinomyces*, accompanied by an increase in *Prevotella*, *Fusobacterium*, and *Porphyromonas* species. Similarly, Bertelsen et al. [51] observed higher microbial diversity in individuals with gingival bleeding compared to those without. Collectively, these findings suggest that insufficient oral hygiene may promote periodontal inflammation through increased microbial load and diversity. Although alpha diversity differed, beta diversity analyses showed no significant differences between oral hygiene groups, indicating that poor hygiene is associated with changes in richness and abundance rather than in overall community composition. This underscores the importance of assessing both within-sample (alpha) and between-group (beta) diversity when characterizing microbial dynamics.

Changes in species diversity are a hallmark of many dysbiotic bacterial states [12]. A recent study in a large cohort of Saudi children reported that the composition of the salivary microbiota differed significantly between high- and low-caries children [52]. A recent systematic review identified Prevotella denticola, Scardoviae Wiggsiae, Streptococcus sobrinus, and Streptococcus mutans as species with higher abundance in adolescents with caries [53]. *Prevotella denticola*, enriched in obese participants, has been implicated in biofilm formation and reported in associated with gingivitis and periodontitis [54]. Similar elevations in *Prevotella* levels have been reported in both obese individuals without periodontitis [55] and those with periodontal disease. Obesity has been associated with alterations in the subgingival microbiota, including an increased abundance of certain pathogenic species that are also linked to periodontal disease. The UW group exhibited significantly higher levels of *Leptotrichia* sp., *Haemophilus* sp., and *Treponema socranskii*, and the genus *Haemophilus* compared to other groups. *Leptotrichia* species have generally been reported in relation to caries [56] and periodontal disease [57]; however, their association with body weight remains unclear. Rahman et al. [58] found Haemophilus to be more abundant in overweight individuals with mild or no periodontitis in Western Asia, while Andrei Bombin et al. [59] identified Actinomyces and Haemophilus as characteristic genera in the salivary microbiota of overweight individuals in the United States. In addition, population-based data from Kuwait suggested that changes in salivary microbiome composition in adolescents with obese or overweight BMI may reflect their susceptibility to oral diseases [33]. Obese patients with chronic periodontitis were also reported to harbor higher levels of pathogenic Treponema socranskii species compared to those with normal body weight [60]. These observations suggest that increased abundance of potentially pathogenic bacteria in individuals with higher BMI may reflect to oral colonization patterns associated with both oral and systemic condition. However, microbiota diversity and composition can be influenced by cultural, dietary, and environmental factors. Therefore, caution should be exercised when making direct comparisons across different populations. The NW group showed elevated levels of *Selenomonas sputigena* and *Mobiluncus* species. Although the genus *Selenomonas* is commonly linked to periodontal disease, *Selenomonas sputigena* has been specifically associated with both periodontitis and gingivitis [61,62]. Interestingly, its abundance has also been observed to be higher in caries-free female adolescents, suggesting variation according to sex- or health-status-dependent variation [63]. Overall, these findings are consistent with the hypothesis that BMI-related differences are associated with microbial composition, which may have implications for oral and systemic health.

The principal limitation of this study is the small sample size (n = 40; 10 individuals per BMI group), which restricts generalizability and reduces statistical power, especially for less abundant taxa. Consequently, the results should be regarded as exploratory and hypothesis-generating rather than conclusive. Moreover, the cross-sectional design does not allow for causal inferences regarding the observed associations. Additional limitations include the reliance on self-reported dietary records, which may be subject to recall bias, the lack of control for menstrual cycle status, and the inability to fully account for the potential influence of regional differences. Potentially influential variables such as socioeconomic status, pubertal status, and the use of oral hygiene products (e.g., fluoride toothpaste, mouthwash) were not assessed. Oral health was evaluated using CPITN and DMFT indices; however, the absence of radiographic imaging, subgingival sampling, plaque scores, or salivary flow measurements may have underestimated periodontal disease severity and microbial diversity. Although the HEI-2015 is a validated dietary assessment tool, it was originally developed for U.S. populations and has not been formally validated in Turkish adolescents, which may limit its applicability. Temporal variability of the salivary microbiota (e.g., diurnal or seasonal fluctuations) was not assessed. Finally, as participants were recruited from one region, potential geographic and cultural differences could not be fully captured, and the restriction to an adolescent age group further limits generalizability. Future research incorporating larger and more diverse cohorts, multi-center participation, and longitudinal or interventional designs will be essential to confirm and extend these observations.

## 5. Conclusions

This study found that the salivary microbiota of healthy Turkish adolescents differs in diversity and taxonomic composition across BMI categories, with underweight individuals exhibiting greater species richness than their obese counterparts. Specific bacterial taxa were enriched in distinct BMI groups, and diet quality (higher consumption of whole fruits, whole grains, and plant-based proteins) was positively associated with beneficial bacterial phyla. Poor oral hygiene correlated with increased microbial richness, although overall community structure remained stable.

To our knowledge, this is the first study to simultaneously evaluate BMI, dietary quality, oral hygiene, and DMFT in relation to the salivary microbiota in adolescents. The evidence from this study suggests that dietary habits, body composition, and oral health parameters are associated with variation in salivary microbiota. These findings may help inform the development of targeted prevention strategies and personalized nutritional approaches. Future research should employ longitudinal or interventional designs to clarify the causal pathways linking diet, BMI, and oral microbiota.

## Figures and Tables

**Table 1:** Demographic and socioeconomic characteristics for the entire sample and in groups based on their BMI z score classification.

Variables ^†^	All Participants (n = 40)	BMI Classification				
		Underweight (n = 10)	Normal Weight (n = 10)	Overweight (n = 10)	Obese (n = 10)	*p*
Age (years)	15.0 ± 0.7	15.0 ± 0.9	14.9 ± 0.6	15.1 ± 0.8	14.9 ± 0.6	0.837 *
Waist circumference (cm)	74.5 ± 13.2	60.9 ± 4.9 ^a^	67.1 ± 4.4 ^a^	80.1 ± 8.7 ^b^	90.1 ± 8.4 ^c^	**<0.001** *
Percentage body fat (%)	24.4 ± 10.6	12.3 ± 5.1 ^a^	22.1 ± 5.9 ^b^	26.7 ± 7.3 ^b^	36.5 ± 5.8 ^c^	**<0.001** *
Gender						
Female	23 (57.5)	5 (50.0)	7 (70.0)	5 (50.0)	6 (60.0)	0.771 **
Male	17 (42.5)	5 (50.0)	3 (30.0)	5 (50.0)	6 (40.0)	
Mother’s educational status						
High school and below	31 (77.5)	5 (50.0)	9 (90.0)	9 (90.0)	8 (80.0)	0.104 **
University and over	9 (22.5)	5 (50.0)	1 (10.0)	1 (10.0)	2 (20.0)	
Father’s educational status						
High school and below	25 (62.5)	5 (50.0)	7 (70.0)	6 (60.0)	7 (70.0)	0.759 **
University and over	15 (37.5)	5 (50.0)	3 (30.0)	4 (40.0)	3 (30.0)	

Abbreviations: BMI, body mass index. ^†^ Values are presented as mean ± standard deviation (SD) for continuous variables and number (n) with percentage (%) for categorical variables. * One Way ANOVA Test; ^a–c^ Superscript letters indicate significant differences between groups. ** Chi-square test. Bold *p*-values signify statistically significant associations (*p* < 0.05).

**Table 2:** Oral hygiene and oral health practices in the entire sample and in groups based on their BMI classification.

Variables ^†^	All Participants (n = 40)	BMI Classification				
		Underweight (n = 10)	Normal Weight (n = 10)	Overweight (n = 10)	Obese (n = 10)	*p* *
Tooth brushing frequency						
Sometimes	13 (32.5)	2 (20.0)	3 (30.0)	4 (40.0)	4 (40.0)	0.542
1 per day	13 (32.5)	3 (30.0)	3 (30.0)	4 (40.0)	3 (30.0)	
2 per day	11 (27.5)	5 (50.0)	3 (30.0)	2 (20.0)	1 (10.0)	
3 per day	3 (7.5)	0 (0.0)	1 (10.0)	0 (0.0)	2 (20.0)	
Tooth brushing time						
<1 min	7 (17.5)	2 (20.0)	1 (10.0)	2 (20.0)	2 (20.0)	0.608
2–3 min	25 (62.5)	7 (70.0)	7 (70.0)	7 (70.0)	4 (40.0)	
>3 min	8 (20.0)	1 (10.0)	2 (20.0)	1 (10.0)	4 (40.0)	
Frequencies of Dental Visits						
When having dental problems	33 (82.5)	7 (70.0)	9 (90.0)	10 (100.0)	7 (70.0)	0.541
Every 6 months	5 (12.5)	2 (20.0)	1 (10.0)	0 (0.0)	2 (20.0)	
Once a year	2 (5.0)	1 (10.0)	0 (0.0)	0 (0.0)	1 (10.0)	
DMFT						
Low	24 (60.0)	4 (40.0)	5 (50.0)	9 (90.0)	6 (60.0)	0.394
Moderate	7 (17.5)	3 (30.0)	2 (20.0)	0 (0.0)	2 (20.0)	
High	9 (22.5)	3 (30.0)	3 (30.0)	1 (10.0)	2 (20.0)	
CPITN						
Good OH	14 (35.0)	2 (20.0)	4 (40.0)	3 (30.0)	5 (50.0)	0.532
Poor OH	26 (65.0)	8 (80.0)	6 (60.0)	7 (70.0)	5 (50.0)	
Deviation						
Yes	20 (50.0)	4 (40.0)	7 (70.0)	4 (40.0)	5 (50.0)	0.494
No	20 (50.0)	6 (60.0)	3 (30.0)	6 (60.0)	5 (50.0)	
Occlusion						
Class I	29 (72.5)	9 (90.0) ^a^	4 (40.0) ^a^	9 (90.0) ^a^	7 (70.0) ^a^	**0.035**
Class II	9 (22.5)	1 (10.0) ^a,b^	6 (60.0) ^b^	0 (0.0) ^a^	2 (20.0) ^a,b^	
Class III	2 (5.0)	0 (0.0) ^a^	0 (0.0) ^a^	1 (10.0) ^a^	1 (10.0) ^a^	

Abbreviations: BMI, body mass index; CPITN, community periodontal index of treatment needs; DMFT, decayed, missing, and filled permanent teeth; OH, oral hygiene. ^†^ Values are presented as number (n) and percentage (%). * Chi-square test, Bold *p*-values signify statistically significant associations (*p* < 0.05). Different letters (a, b) denote significant differences (*p* < 0.05).

## Data Availability

The original contributions presented in this study are included in the article. Further inquiries can be directed to the corresponding author.

## Abbreviations

The following abbreviations are used in this manuscript:

ASVs	Amplicon Sequence Variants
BMI	Body mass index
CPITN	Community periodontal index of treatment needs
DMFT	Decayed-missing-filled teeth
HEI	Healthy Eating Index
LEfSe	Linear discriminant analysis Effect Size
NW	Normal weight
OB	Obese
OH	Oral hygiene
OW	Overweight
PBS	Phosphate-Buffered Saline
PCoA	Principal Coordinate Analysis
PCR	Polymerase chain reaction
SPSS	Statistical Package for Social Sciences
TNs	Treatment need scores
UW	Underweight
WHO	World Health Organization
